# Prolene (mesh) bulbourethral sling in male incontinence

**DOI:** 10.4103/0970-1591.30262

**Published:** 2007

**Authors:** Rakesh Kapoor, Kamal Jeet Singh, Amit Suri, Pratipal Singh, Anil Mandhani

**Affiliations:** Department of Urology, Sanjay Gandhi Post Graduate Institute of Medical Sciences, Lucknow, India

**Keywords:** Bulbar urethral sling, post prostatectomy incontinence, urinary incontinence

## Abstract

**Objective:**

We present our preliminary results of bulbar urethral sling (single bolster) in treatment of postprostatectomy urinary incontinence (PPUI).

**Materials and Methods:**

From May 2003 to June 2005, six patients with postprostatectomy urinary incontinence (transurethral resection of prostate in five patients and after open prostatectomy in one patient) underwent prolene mesh bulbar urethral sling surgery. Preoperative evaluation included physical examination, neurological assessment, stress cystogram and urethrocystoscopy. Urodynamic evaluation was done in all patients for abdominal leak point pressure and ruling out bladder pathology.

**Results:**

Urodynamic studies did not demonstrate bladder instability in any patient. Mean abdominal leak point pressure was 43cm of water (range 26–80 cm of water). Mean duration of hospital stay was 3.2 days. Follow-up ranged from 6–22 months. Four patients out of six patients were completely dry till their last follow-up. One patient developed mild stress incontinence after one year of the surgery and required use of one to two pads per day. Mean pad use after surgery was 0.6 pads per day in comparison to mean pad usage of 6.4 pads per day preoperatively. One patient was over-continent after the procedure and required clean intermittent catheterization till last follow-up (six months). Mean cost of the procedure was $ 350+15.

**Conclusion:**

Prolene bulbar urethral sling (single bolster) is an economically effective option in patients with postprostatectomy urinary incontinence.

Postprostatectomy urinary incontinence (PPUI) is an uncommon complication, whether they are performed by the transurethral or transvesical route. It is estimated that approximately 1% of patients who undergo these surgeries develop urinary incontinence.[1] While detrusor instability may have a role, malfunction of the internal sphincter appears to be the reason for incontinence.[2,3] Mild degrees of urinary incontinence may be improved or cured with Kegel exercises and pharmacotherapy but more severe incompetence warrants a more aggressive approach - bolster the sphincter mechanism. The accepted surgical techniques for management of postprostatectomy urinary incontinence are - transurethral injection and artificial sphincter insertion.

Transurethral injections are technically simple and a minimally invasive method but they are expensive and many patients may require multiple injections. The various substances most often used include bovine collagen, autologous fat, texturized silicone and more recently, pyrolitic carbon stands out. In developing countries injectables are marketed at high costs, making their routine use unfeasible, especially when one considers the frequent need of repeat injections for obtaining and maintaining the results. Significant improvement in urinary leaks lies around 30–60%, with a follow-up longer than 24 months, being variable according to the substance employed.[4]

The artificial sphincter is successful at achieving continence but revisions are often necessary because of erosion and mechanical problems; manual dexterity is required to operate the device.[5–7] Artificial sphincters are available at high costs in the developing countries which make their use limited.

Sling procedures have been used for many years to treat women with intrinsic sphincter deficiency. Long-term continence rates approach 90% in this group of patients.[8,9] Male urethral slings using both autologous and synthetic material have been described in the literature.[10,11] The principle is common to all these procedures i.e. bolster to the bulbar urethra either by suspension from the rectus fascia or by use of bone anchors. The procedure allows physiological voiding and is cheaper than the artificial urinary sphincter. We had published our previous result using single Dacron bolster in PPUI patients.[12] Now we present our initial experience using prolene mesh as single bolster in patients with PPUI.

## MATERIALS AND METHODS

From March 2003 to June 2005 six male patients with urinary incontinence postprostatectomy underwent correction of their incontinence using our modified bulbar urethral sling using prolene mesh (Johnson and Johnson, Aurangabad, India). Of these six patients, five had incontinence following transurethral resection of prostate and one had incontinence following open prostatectomy. All these patients were incontinent for more than one year after their primary surgery (prostatectomy). Preoperative evaluation included physical examination, neurological assessment, stress cystogram and urethrocystoscopy. Urodynamic evaluation included uroflow, cystometrogram and abdominal leak point pressure measurement.

Under general or epidural anesthesia the patient was placed in lithotomy position, a midline incision was made in the perineum and the bulbar urethra was dissected. Following mobilization of the bulbar urethra the perineal diaphragm and membranous urethra were identified. A 3-cm incision was made in the abdomen in the midline just above the pubic symphysis and deepened to the rectus sheath. A Stamey needle was placed on the anterior rectus fascia lateral to the midline on one side of the superior border of the symphysis pubis. The needle was directed along the inner surface of the pubic bone parallel to the periosteum and towards the perineum. An index finger was placed at the inferior wall of the urogenital diaphragm between the membranous urethra and bulbospongiosus muscle. The needle was then penetrated alongside the membranous urethra and through the endopelvic fascia guided by the surgeon's index finger. The position of the needle was checked endoscopically using 30-degree telescope to ensure that needle had not perforated the urethra. A 70-degree telescope was used to ensure that there was no inadvertent passage through the bladder during the needle passage.

**Figure 1:**
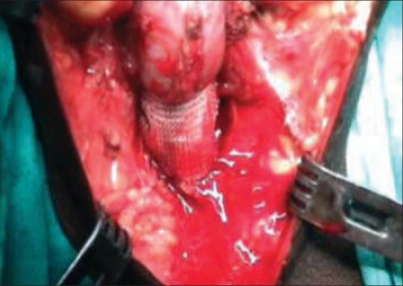
Tailored mess on the urethra after sutures are pulled

A no. 1 prolene thread was threaded through over the needle and pulled up in the suprapubic region leaving the needle and nonneedle end of the suture lying in the perineum on one side of the membranous urethra. The same procedure was repeated on the other side. A prolene mesh (5 × 2 cm) was taken doubled on its own. Prolene suture was passed through the mesh in continuous fashion. The length of the mesh (distance between the two exit points of the Stamey needle in the perineal diaphragm) was tailored to maintain tension on the bulbar urethra. The prolene sutures were pulled to place the mesh over the bulbar urethra at the level of perineal membrane [Figure 1]. Since the exit points of the needle in the perineal membrane are fixed, the tension of the sling against the bulbar urethra was adjusted by tailoring the length of the mesh between these two exit points.

Each prolene suture was first passed through the rectus sheath and knotted and thereafter tied to each other with tension. The suprapubic wound was closed. A catheter was passed to check the final urethral patency and ease of catheterization. The bulbospongiosus muscle was closed with running sutures and the wound was closed after adequate hemostasis had been ensured. A suprapubic catheter drained the urine. Adequate antibiotic coverage was given preoperatively and postoperatively.

The patient was given voiding trial after the third postoperative day and the suprapubic catheter was removed as soon as the postvoiding residual urine volume was less than 50 ml. Postoperatively, in the follow-up, upper tract monitoring was carried out by abdominal ultrasonography. The voiding pattern was assessed by uroflowmetery and postvoid residual urine measurement taken once a month for three months and then once every three months. Detailed urodynamic studies were not performed as the postvoid residual volume was less than 50ml and none of the patients developed symptoms suggestive of bladder instability.

## RESULTS

Mean age of patients was 56.5 + 6.5 years. Mean duration of incontinence after surgery was 2.1 years (1.3-4.6 years). Mean pad usage was 6.4 pads per day preoperatively. Urodynamic studies did not demonstrate bladder instability in any patient. The mean abdominal leak point pressure was 42 cm of water (range 26-80 cm of water). None of our patients had a perineal wound infection or erosion. Follow-up ranged from 6-22 months. Four patients were completely dry till their last follow-up while one patient complained of mild stress urinary incontinence requiring use of one to two pads per day. One patient was over-continent after the procedure and required clean intermittent catheterization till last follow-up. This patient refused further evaluation and was lost to follow-up after a period of six months. Complete continence was achieved in 67% patients (four out of six). Socially acceptable continence was achieved in 83.3% (five out of six). No erosion and recurrent urinary infections were reported in this group of patients. Mean cost of the procedure was $350 +15 (this includes hospital stay, drugs, operation charges etc.).

## DISCUSSION

Male urinary incontinence, due to sphincteric insufficiency, following endoscopic resection of prostate is a feared complication as it severely affects the quality of life. In men with sphincteric insufficiency the slings compress the bulbar urethra and prevent the leakage of urine. Preoperatively all patients should have a urodynamic evaluation to document the normal detrusor muscle contraction. Stenosis of the urethra or bladder neck, bladder with low capacity and/or low compliance are relative contraindications for the primary procedure.[13–15]

Schaeffer described a modified bulbourethral sling procedure using three bolsters to augment urethral resistance in postradical prostatectomy incontinence.[16] We performed modified bulbar urethral sling procedure in patients having intrinsic sphincter deficiency after transurethral resection of the prostate using a single broad base sling.

We believe that synthetic materials - polypropylene, Dacron, PTTE mesh, etc – are easy to handle and, since the pressure over the urethra is low and largely extended, the risk of urethral erosion is minimal. Our technique is modification of our previously described technique in which we used Dacron patch to bolster.[12] The advantages of the prolene mesh were easy availability, low cost and presence of pores which allowed the fibrous tissue growth through it. We feel that dissecting the bulbospongiosus makes needle placement easier on both sides of the membranous urethra and chances of over-continence are lessened. Technically male urethral sling is an easy procedure but careful attention is required.

Infection is a major concern because of perineal bacterial flora and use of synthetic grafts. We did not encounter any significant infection in our patients. We usually employ antibiotics effective against both aerobic and anaerobic bacteria. Intraoperative antibiotic irrigations are routinely performed during the course of the surgery.

**Figure 2:**
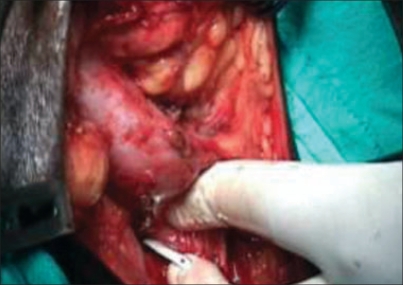
Exit of Stamey needle on one side of the urethra

We maintain utmost care in maintaining sterility during mesh handling. Cystourethroscopy was routinely performed during needle passage or immediately after needle passage to rule out inadvertent passage through the urinary tract. During needle passage care was taken to maintain contact with the pubic bone at all times and to exit the Stamey needle through the perineal membrane on one side of the membranous urethra under finger guidance [Figure 2]. Prolene mesh placement between the bulbospongiosus muscle and bulbar urethra has many advantages: it augments tension on the bulbar urethra and facilitates a secure placement of the prolene sling and decreases the chances of perineal erosion. None of our patients had urethral erosion due to the sling till their last follow-up. Moreover, the bolster should provide adequate compression of the bulbar urethra.

**Figure 3:**
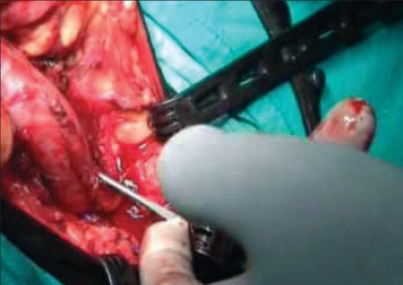
Exit of Stamey needle on the other side of the urethra

The tension on the urethra depends on the length of the sling between the two exit points of the Stamey needle in the perineal membrane [Figure 3], not on the prolene suture passed suprapubically. This latter suture only helps in holding the mesh but plays a minimal role in achieving continence. Five of our patients had good continence after the surgery, while one patient had retention of urine after the surgery. This patient was using penile clamp in the preoperative period to maintain continence. This had led to the dilatation of the proximal urethra, which was also documented on the preoperative retrograde urethrogram. We observed puckering of the urethral mucosa on intraoperative urethroscopy after the sling was placed. We presume this puckering of the mucosa may have led to the increased urethral resistance leading to retention of urine though we could not document it as the patient refused further evaluation and was lost to follow-up.

Artificial urinary sphincter placement has emerged as the gold standard for the treatment of severe postprostatectomy incontinence. However, despite advances in design, malfunctions and erosions still occur.[15,16] In addition, a successful operation requires manual dexterity. Because of the simple material used to make the bolster, the cost of the bulbourethral sling procedure is significantly lower than that required for the insertion of the artificial urinary sphincter. There are many differences between the artificial urinary sphincter and the bulbourethral sling. While the artificial urinary sphincter is a prosthetic surrogate sphincter that circumferentially compresses the urethra, the bulbar urethral sling compresses only a part of the circumference of the urethra. The biophysical difference might produce significant physiological difference in efficacy. The idea behind the sling is that the urethra will be compressed against the sling during changes in the abdominal pressure.

The prolene bulbar urethral sling is an economically viable procedure in the developing country scenario in comparison to the artificial sphincter. The mean cost of the procedure in our group of patients was ∼$350 dollars in comparison to $4000 for the AMS 800 artificial sphincter (source: AMS website).

## CONCLUSIONS

Prolene bulbar urethral sling is a safe, effective and viable option in managing patients with PPUI.
